# Exploration of the relationships between immune cells, metabolic mediators, and atrial fibrillation: A bidirectional Mendelian randomization study

**DOI:** 10.1097/MD.0000000000041348

**Published:** 2025-03-14

**Authors:** Hongliang Huang

**Affiliations:** a Affiliated Hospital of Jinggangshan University, Ji’an, Jiangxi, China.

**Keywords:** atrial fibrillation, immune cells, Mendelian randomization, metabolic mediators

## Abstract

Studies have shown a close correlation among immune cells, plasma metabolites, and atrial fibrillation (AF). However, it is not clear if this association is related, which we used Mendelian randomization (MR) to investigate. We analyzed the association between immune cells, plasma metabolites, and AF by using summarized data from genome-wide association studies. Among them, we explored the associations between immune cells and AF by using bidirectional MR analysis. Combined with mediation analysis and multivariable MR, we further identified potential mediating plasmic metabolites. Results shows that causal relationships between 8 immune cell phenotypes and AF were identified with all 8 exhibiting reverse causality. Furthermore, 22 plasma metabolites have a causal relationship with AF. In addition, 2 immune cell phenotypes including CD25 on IgD + CD38dim and CX3CR1 on CD14 + CD16-monocyte, which were found to have causal relationships with 4 plasma metabolites, including 4-acetamidobutanoate levels, Octadecanedioylcarnitine (C18-DC) levels, Linolenate [alpha or gamma; (18:3n3 or 6)] levels, and N-acetyl-aspartyl-glutamate levels, which might be mediators. Ultimately, only 4-acetamidobutanoate levels, CD25 on IgD + CD38dim, and AF did appear to function as mediators (*P*-value = .030 < .05). In conclusion, immune cells and plasma metabolites are causally associated with AF. We have identified that 4-acetamidobutanoate levels appear to mediate the pathway linking CD25 on IgD + CD38dim to AF. This finding provides a new perspective for the early prevention and diagnosis of preatrial AF.

## 1. Introduction

Atrial fibrillation (AF), characterized by the irregular and abnormal contractions of the atrial cardiomyocytes, is the most common type of cardiac arrhythmia.^[1]^ Based on persistence, AF could be classified into paroxysmal AF, persistent AF, long-standing persistent AF, and permanent AF.^[2]^ The morbidity for AF increases with the presence and severity of underlying disease, including congestive heart failure, valve disease, hypertension, diabetes mellitus, and obesity.^[3]^ There are also differences in sex, age, geographical regions, and socioeconomic status among AF patients. Epidemiological data indicate that AF occurs in fewer than 1% of persons aged 60 to 65 years but in 8% to 10% of those older than 80 years. Men have a higher prevalence than women, irrespective of socioeconomic status.^[4]^ The patient map shows that Western Europe and North America have higher prevalence and generally lower prevalence in South Asia, Oceania, and the Middle East.^[5]^ Patients with AF are steadily rising owing to population aging, advancement of urban life, and dietary habits. In meta-analyses, AF is also associated with increased risk of multiple negative consequences, including cognitive impairment or dementia (1.5-fold), stroke (2.4-fold), myocardial infarction (1.5-fold), sudden cardiac death (2-fold), heart failure (5-fold), chronic kidney disease (1.6-fold), and peripheral artery disease (1.3-fold).^[6]^ Globally, AF is associated with a loss of 6.0 million disability-adjusted life-years, a measure of the overall burden of disease, in 2017, and this number continues to increase.^[7]^ Hence, AF constitutes a significant clinical and social burden worldwide and is associated with substantial morbidity and mortality.

The pathogenesis of AF is complex, involving abnormal anatomical structure, unregulated ion channels and proteins, and the interaction between cells in the conduction system, cardiomyocytes, fibroblasts, and the immune system.^[8]^ There is an increasing evidence that the immune cell abnormality and dysfunction play an important role in the occurrence and development of AF, which may provide a new insight for research and therapeutic strategies. Researchers came up with the concept of immune remodeling, meaning that the changes in the immune system in AF as another form of immune remodeling.^[9]^ It refers to an immune network composed of recruitment and activation of immune cells and secretion of immune molecules induced by various factors in the AF.^[10]^ Notably, immune remodeling is not only limited to the atria unlike remodeling in atrial, but also affects the peripheral circulation, suggesting that AF should be identified as a systemic disease from the perspective of immune imbalance.^[11]^ A study in 2023 has revealed a causal relationship between peripheral lymphocytes, especially the CD4 + T cell counts, and AF.^[12]^ However, the evaluation in related research of the immune system in AF is difficult and limited at present stage. Most investigations only evaluate the order of magnitude and existence, but it is difficult to evaluate and quantify its functions and crosslinking reaction. Therefore, researches focusing on immunology may help raise awareness of AF.

The massive metabolites in plasma affect immune regulation. Various microbial-derived factors, whether produced or transformed by microorganisms, are major participants in the dialogue between the body and immune cells.^[13]^ In general, a large proportion of metabolites participate in the immune function without causing harmful immune responses. When the microbiome changes and the intestinal barrier breaks down, the compounds of metabolites cause chronic inflammatory responses and cascading effects along with the circulation.^[14]^ In recent years, several observational studies have found lysophosphatidylcholine 18:1 and 18:2, monoglyceride 18:2, and sphingomyelin 28:1 to be related to incident coronary heart disease,^[15]^ sphingomyelin 32:1 to be associated with incident stroke,^[16]^ and urobilin and sphingomyelin 30:1 to be linked to incident heart failure,^[17]^ showing plasmic metabolomics has the ability to discover novel biomarkers for cardiovascular disease. In addition, changes in derivatives and metabolites caused by dysregulation of gut microbiota also have also been shown to contribute to the occurrence and exacerbation of AF, such as chenodeoxycholic acid,^[18]^ trimethylamino oxide,^[19]^ lipopolysaccharide,^[20]^ and etc. Given the systemic role of immune response and plasma metabolites, more valuable studies will help enrich the mechanism and drug development of AF. Nevertheless, there is a deficiency of extensive longitudinal studies to substantiate the causal relationship between immune cells, metabolic mediators, and AF and their respective mediation proportions remain unclear. Moreover, the role of plasma metabolites in these associations can only be partially corroborated due to the complexity of immune cells, plasma metabolites, and the AF.

With the rapid development of life science and computer technology, Mendelian randomization (MR) has made it possible for exploring causal effects from modifiable exposure on complex diseases.^[21]^ Based on the law of genetics, which is referred to independent segregation of genetic alleles, genetic variants, commonly single-nucleotide polymorphisms (SNPs), are used as instrumental variables (IVs) for the putative risk factor in MR analysis.^[22]^ The risk of confusion is reduced by producing subgroups with similar clinical characteristics, which is similar to the random allocation in randomized controlled trials. Because it could be conducted using existing large-scale genome-wide association studies (GWAS) data, MR studies have ability to evaluate numerous sample sizes and long-term follow-up outcomes. In addition, for certain tests that are not available in clinical trials because of any reasons, researchers can explore exposure to adverse effects through MR analysis. In contrast to costly and demanding randomized controlled trials, MR analysis helps to reflect the life-long impact of risk factors because of the fixed genetic variation. Therefore, MR studies are a valuable design that can overcome some of the limitations and problems faced by traditional observational studies and randomized controlled trials.

**Figure 1.**
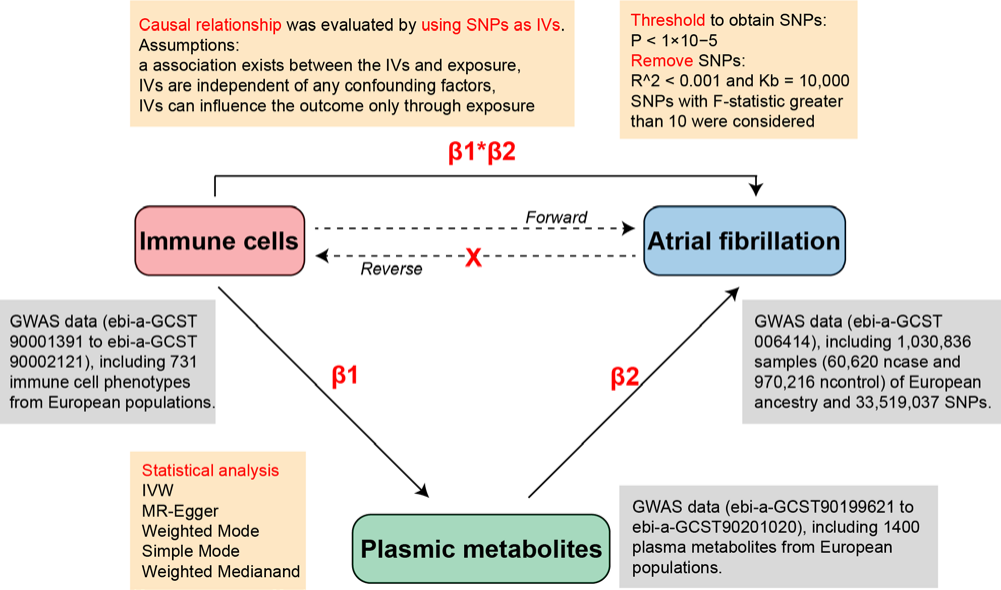
The schematic diagrams depicting the research.

Our study may be the first to conduct a bidirectional MR analysis with 2 samples and a mediation analysis using data from recent GWAS to investigate the potential role of metabolites as mediators in the pathway from immune cells to AF. This helps to deepen researchers’ understanding of the physiological and pathological mechanisms of AF and to advance our understanding of this cardiovascular disease from an immunological basis. The schematic diagrams depicting the research is shown in Figure 1.

## 2. Methods

### 2.1. Study design

Four-steps MR analyses have been used to assess the causal relationship between immune cell phenotypes and AF. Firstly, our study employed bidirectional MR to evaluate the causal relationship between 731 immune cell phenotypes and AF via forward and reverse MR. Secondly, the causal effects of 731 immune cell phenotypes on 1400 plasma metabolites were examined (β1). Next, the causal effects of 1400 plasma metabolites on AF were examined (β2). Finally, multivariable MR approaches to elucidate the plasmic metabolites-mediated effects of immune cells on AF were calculated (β1×β2). Additionally, by employing SNPs as IVs, we can leverage the MR approach to infer causality without the confounding factors that often plague observational studies, such as reverse causation or unmeasured confounding. Moreover, the following 3 fundamental assumptions must be satisfied: a strong association exists between the IVs and exposure, IVs are independent of any confounding factors, and IVs can influence the outcome only through exposure (MR: Methods for Causal Inference Using Genetic Variants: CRC Press).

### 2.2. Data sources

We extracted data on associations of AF from the IEU OpenGWAS project (https://gwas.mrcieu.ac.uk/), utilizing the dataset identified as ebi-a-GCST006414, which including 1030,836 samples (60,620 ncase and 970,216 ncontrol) of European ancestry and 33,519,037 SNPs. Genetic data of 731 immune cell phenotypes (ebi-a-GCST90001391 to ebi-a-GCST90002121) and 1400 plasma metabolites (GCST90199621–GCST90201020) were obtained from previous GWAS study,^[23,24]^ and all samples from European populations.

### 2.3. IVs selection

We used *P* < 1×10^−5[25,26]^ as a threshold to obtain SNPs associated with immune cell phenotypes and plasma metabolites, while using the criteria of *R*^2^ < 0.001 and Kb = 10,000^[27]^ to remove SNPs with linkage disequilibrium. SNPs with minor allele frequency ≤ 0.01 were removed. The F-statistic for each SNP was calculated to determine its statistical strength in relation to exposure. The F-value is calculated using the formula: F = [(N−K−1)*R*^2^]/[K(1−*R*^2^)], where *R*^2^ represents the proportion of variance explained by genetic variation, N denotes the sample size, and K indicates the number of SNPs included. The *R*^2^ value is computed using the formula *R*^2^ = 2 × β^2^× EAF × (1 − EAF). F-statistic less than 10 warns of potential weak instrument bias, which can lead to inconsistent estimates. Consequently, the F-statistic need greater than 10.^[28,29]^ To reduce the bias resulting from weak IVs, only SNPs with F-statistic >10 were considered for further screening for disease-related immune cell phenotypes and plasma metabolites. Meanwhile, palindromic SNPs and SNPs which strongly associated with outcome-associated were excluded from the study.

### 2.4. Statistical analysis

Five MR methods, including inverse variance weighted (IVW), MR-Egger, weighted mode, simple mode, and weighted median methods, were employed to evaluate the potential causal link. The primary IVW method for MR analysis is effective at estimating causal effects and addressing potential heterogeneity.^[21,30]^ A *P*-value below .05 in the IVW method was taken as evidence of a causal relationship,^[31]^ while the other 4 methods were used for additional analysis.^[32]^ To improve the reliability of our results, we conducted a sensitivity analysis by applying the “leave-one-out” method. To identify the presence of heterogeneity, the Cochran *Q* test was performed, with a Cochran-*Q* derived *P*-value of less than .05 indicating significant heterogeneity. Horizontal pleiotropy was assessed using MR-Egger intercepts. Furthermore, the MR-PRESSO test was utilized to detect pleiotropy and to manually exclude SNPs identified as outliers.^[33,34]^ Data were analyzed by using the TwoSampleMR (TSMR) (version 0.6.5) and MR-PRESSO (version 1.0) packages in R (version 4.4.1). Using the TSMR approach, we calculated the total effect from immune cells to AF, as well as the effects of immune cells on metabolites (β1) and metabolites on AF (β2). We then calculated the mediating effect as the product of β1 and β2. The direct effect was determined by subtracting the mediating effect from the total effect.^[35]^

## 3. Results

### 3.1. Genetic causality between immune cells and AF

**Figure 2.**
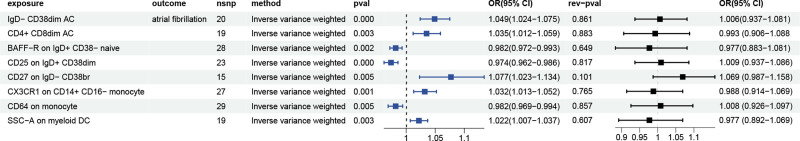
Forest plots of the causal relationship between immune cells and AF. AF = atrial fibrillation.

Utilizing a multistep selection of IVs, we ultimately identified 13,318 SNPs associated with immune cells. The minimum F-value for these genetic instruments was recorded at 19.53 indicating the research was not susceptible to weak IVs. There were significant associations between 8 immune cell phenotypes and AF via the IVW method, including IgD− CD38dim AC, CD4 + CD8dim AC, BAFF-R on IgD + CD38− naive, CD25 on IgD + CD38dim, CD27 on IgD− CD38br, CX3CR1 on CD14 + CD16− monocyte, CD64 on monocyte, and SSC-A on myeloid DC. Taken into account the potential problem of reverse causation, we performed a reverse MR analysis with AF as the exposure and 8 immune cell phenotypes as the outcome. Limited evidence was found for the causal effect of AF on any of the 8 immune cell phenotypes in the reverse MR analysis, as evidenced by a reverse *P*-value greater than .05. Additionally, 5 immune cell phenotypes are associated with an increased risk of AF, including IgD- CD38dim AC, CD4 + CD8dim AC, CD27 on IgD- CD38br, CX3CR1 on CD14 + CD16- monocytes, and SSC-A on myeloid dendritic cells (DC). Conversely, 3 immune cell phenotypes are inversely associated with AF, namely BAFF-R on IgD + CD38- naive cells, CD25 on IgD + CD38dim cells, and CD64 on monocytes. We utilized Cochran *Q* test to assess for heterogeneity and the MR-Egger intercept test to evaluate pleiotropy, both serving as statistical measures of evidence. The findings revealed that all *P*-values obtained from Cochran *Q* test were above the .05 threshold, pointing towards no heterogeneity. Simultaneously, the MR-Egger intercept test did not detect any significant horizontal pleiotropy across the outcomes (Table 1, Fig. 2).

**Table 1:** Forward and reverse MR analyses of immune cells and AF.

Exposure	Outcome	Method	nSNP	Beta	SE	*P*-value	rev*P*-value
IgD- CD38dim AC	AF	IVW	20	0.047759992	0.012393675	.000116404	.861456015
CD4 + CD8dim AC	AF	IVW	19	0.034555867	0.011788925	.003376397	.882845802
BAFF-R on IgD + CD38- naive	AF	IVW	28	-0.01770349	0.005701584	.001902709	.649104966
CD25 on IgD + CD38dim	AF	IVW	23	-0.026820625	0.006266071	1.87E-05	.817419482
CD27 on IgD- CD38br	AF	IVW	15	0.07396928	0.02622544	.004794644	.100678112
CX3CR1 on CD14 + CD16- monocyte	AF	IVW	27	0.03188405	0.009489955	.000780079	.76538973
CD64 on monocyte	AF	IVW	29	-0.018427438	0.006585467	.005138864	.857282985
SSC-A on myeloid DC	AF	IVW	19	0.021673913	0.007263571	.002845815	.606697107

AF = atrial fibrillation, IVW = inverse variance weighted, MR = Mendelian randomization, SNPs = single-nucleotide polymorphisms.

### 3.2. Genetic causality between plasmic metabolites and AF

**Figure 3.**
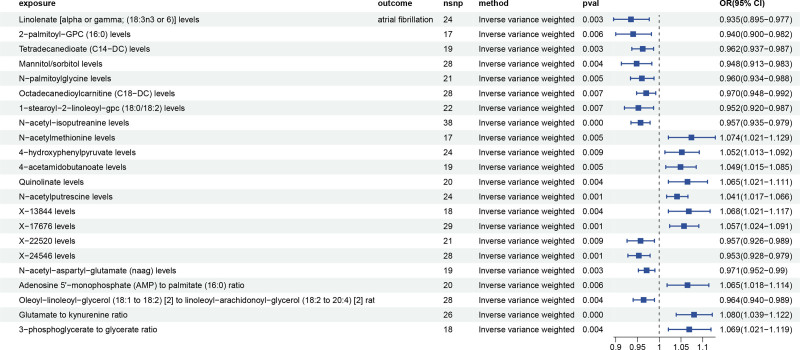
Forest plots of the causal relationship between plasma metabolites and AF. AF = atrial fibrillation.

Through a multistep selection of IVs, we initially identified 29,302 SNPs associated with plasma metabolites. The F-value for the genetic instruments all exceeded the standard threshold of 19.50, indicating that the research was not vulnerable to the influence of weak IVs. There were significant associations between 22 plasma metabolites and AF via the IVW method, including 18 known and 4 unknown metabolites. Among the known metabolites, 8 were identified as potentially increasing the risk of AF, specifically elevated levels of N-acetylmethionine levels, 4-hydroxyphenylpyruvate levels, 4-acetamidobutanoate levels, Quinolinate levels, N-acetylputrescine levels, Adenosine 5’-monophosphate (AMP) to palmitate (16:0) ratio, Glutamate to kynurenine ratio, and 3-phosphoglycerate to glycerate ratio. In contrast, genetically predicted 10 metabolites were identified as lower risk with AF, including Linolenate [alpha or gamma; (18:3n3 or 6)] levels, 2-palmitoyl-GPC (16:0) levels, Tetradecanedioate (C14-DC) levels, Mannitol/sorbitol levels, N-palmitoylglycine levels, Octadecanedioylcarnitine (C18-DC) levels, 1-stearoyl-2-linoleoyl-gpc (18:0/18:2) levels, N-acetyl-isoputreanine levels, N-acetyl-aspartyl-glutamate (NAAG) levels, and Oleoyl-linoleoyl-glycerol (18:1 to 18:2) [2] to linoleoyl-arachidonoyl-glycerol (18:2–20:4) [2] ratio (Table 2, Fig. 3).

**Table 2:** MR analyses of the causal effects between plasma metabolites and AF.

Exposure	Outcome	Method	nSNP	Beta	SE	*P*-value
Linolenate [alpha or gamma; (18:3n3 or 6)] levels	AF	IVW	24	-0.067058013	0.02227208	.002605128
2-Palmitoyl-GPC (16:0) levels	AF	IVW	17	-0.061825938	0.022315639	.005596675
Tetradecanedioate (C14-DC) levels	AF	IVW	19	-0.039048663	0.01306615	.002803172
Mannitol/sorbitol levels	AF	IVW	28	-0.053765396	0.018775648	.004188953
N-Palmitoylglycine levels	AF	IVW	21	-0.040317577	0.01451771	.005484114
Octadecanedioylcarnitine (C18-DC) levels	AF	IVW	28	-0.030960631	0.011576828	.007487157
1-Stearoyl-2-linoleoyl-gpc (18:0/18:2) levels	AF	IVW	22	-0.04874065	0.017934203	.006572781
N-Acetyl-isoputreanine levels	AF	IVW	38	-0.044170238	0.011956965	.000220666
N-Acetylmethionine levels	AF	IVW	17	0.071204732	0.025606137	.005423044
4-Hydroxyphenylpyruvate levels	AF	IVW	24	0.050294625	0.019208705	.008836271
4-Acetamidobutanoate levels	AF	IVW	19	0.048241363	0.017076282	.004727312
Quinolinate levels	AF	IVW	20	0.062786072	0.021589921	.003636093
N-Acetylputrescine levels	AF	IVW	24	0.039988989	0.011967846	.000833663
X-13844 levels	AF	IVW	18	0.06566656	0.022906131	.00414687
X-17676 levels	AF	IVW	29	0.055532972	0.016146305	.000583089
X-22520 levels	AF	IVW	21	-0.044099094	0.016996593	.00947054
X-24546 levels	AF	IVW	28	-0.047952016	0.013827501	.000524595
N-Acetyl-aspartyl-glutamate (NAAG) levels	AF	IVW	19	-0.029182016	0.009957685	.003383054
Adenosine 5’-monophosphate (AMP) to palmitate (16:0) ratio	AF	IVW	20	0.063117	0.022888758	.005823548
Oleoyl-linoleoyl-glycerol (18:1 to 18:2) [2] to linoleoyl-arachidonoyl-glycerol (18:2 to 20:4) [2] ratio	AF	IVW	28	-0.036927707	0.012939381	.004318606
Glutamate to kynurenine ratio	AF	IVW	26	0.076758714	0.019400097	7.60E-05
3-Phosphoglycerate to glycerate ratio	AF	IVW	18	0.066660209	0.023260746	.004159816

AF = atrial fibrillation, MR = Mendelian randomization, SNPs = single-nucleotide polymorphisms.

### 3.3. Mediated MR analysis

To further calculate mediation, we used the TSMR method by MR, utilizing the estimates derived from the IVW analyses. The mediation analyses were conducted building on the immune cells and plasma metabolites that had already been identified. We performed an MR analysis from immune cell phenotypes to plasma metabolites, using the 22 plasma metabolites as outcome and the 8 immune cell phenotypes as exposure. The impact size β1 from immune cell phenotypes to plasma metabolites was obtained from this study, which also showed causal links between 2 immune cell phenotypes and 4 plasma metabolites.

**Figure 4.**
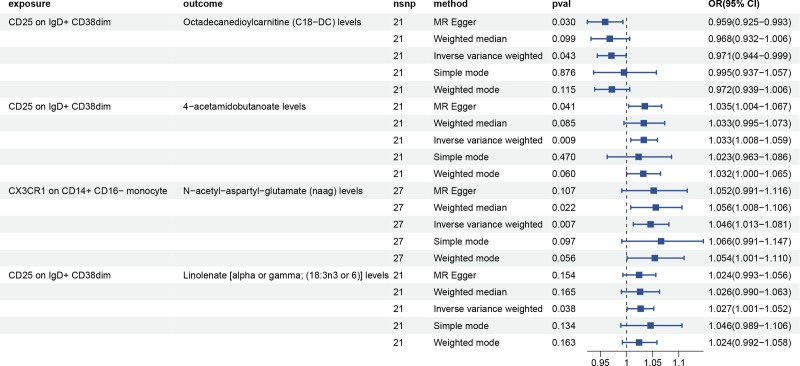
Forest plots of the causal relationship between immune cells and plasma metabolites.

According to our study, we have identified a positive correlation between the expression of CX3CR1 on CD14 + CD16- monocyte and NAAG levels. Additionally, our findings suggest that a single immune cell phenotype can be causally related to multiple metabolites. For example, CD25 on IgD + CD38dim have correlation with 4-acetamidobutanoate levels, Octadecanedioylcarnitine (C18-DC) levels, and Linolenate [alpha or gamma; (18:3n3 or 6)] levels. There is a positive correlation between CD25 on IgD + CD38dim and 4-acetamidobutanoate levels, a negative correlation between CD25 on IgD + CD38dim and Octadecanedioylcarnitine (C18-DC) levels, a positive correlation between CD25 on IgD + CD38dim and Linolenate [alpha or gamma; (18:3n3 or 6)] levels. Then we used 4 plasma metabolites as exposure and AF as outcome to conduct MR analysis. The results were revealed no evidence of pleiotropy and unbiased SNPs (*P* > .05). This analysis facilitated the estimation of the effect size β2 from metabolites to AF. Subsequently, the overall effect from immune cells to AF was calculated (Table 3, Fig. 4).

**Table 3:** MR analyses of the causal effects between immune cells and plasma metabolites.

Exposure	Outcome	Method	nSNP	Beta	SE	*P*-value
CD25 on IgD + CD38dim	4-Acetamidobutanoate levels	IVW	21	0.032628447	0.012486839	.008974422
CD25 on IgD + CD38dim	Octadecanedioylcarnitine (C18-DC) levels	IVW	21	-0.0294379	0.0145238	.042675389
CD25 on IgD + CD38dim	Linolenate [alpha or gamma; (18:3n3 or 6)] levels	IVW	21	0.02616652	0.012587362	.037636382
CX3CR1 on CD14 + CD16- monocyte	N-Acetyl-aspartyl-glutamate (NAAG) levels	IVW	27	0.045314168	0.016773515	.006902109

MR = Mendelian randomization, SNPs = single-nucleotide polymorphisms.

### 3.4. Mediation analysis

**Figure 5.**
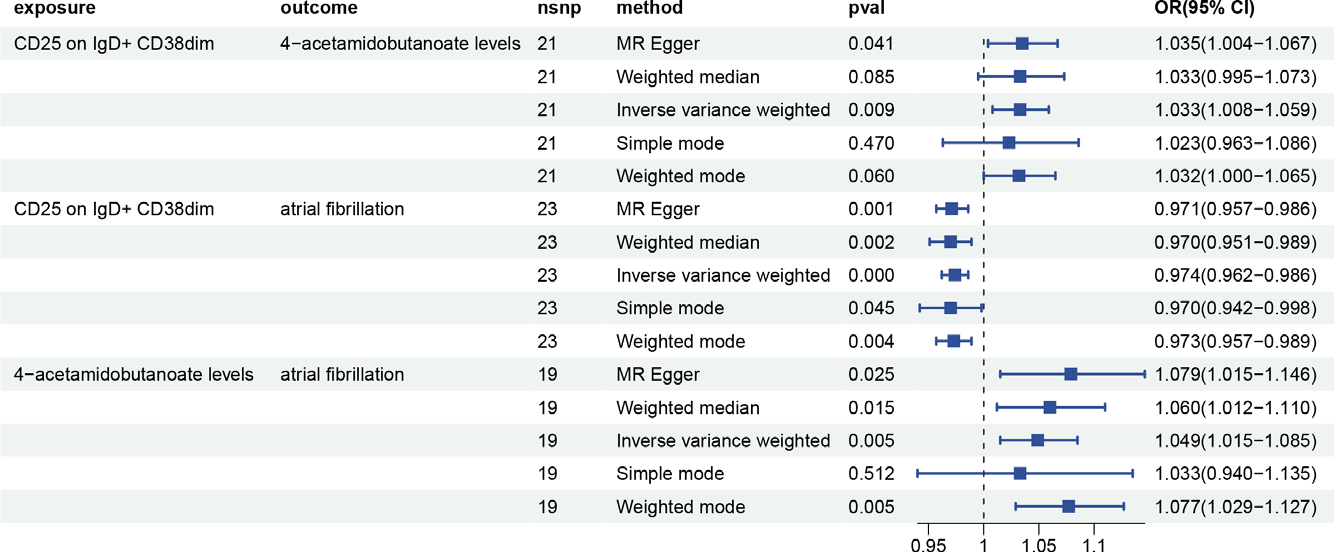
Target plasma metabolite mediate the causal relationship between immune cell and AF. AF = atrial fibrillation.

**Figure 6.**
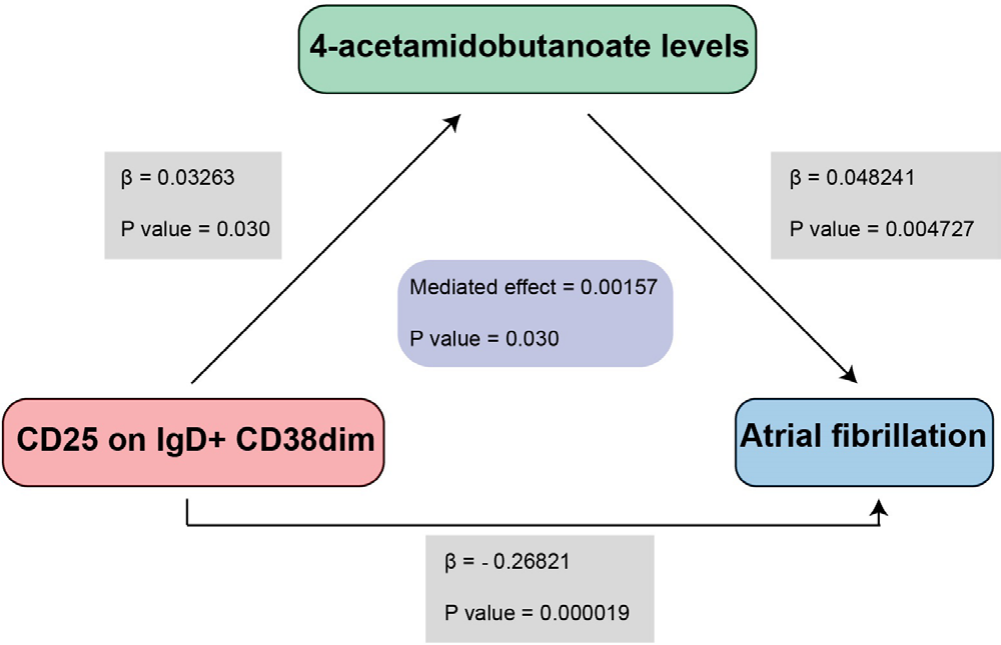
Results and thumbnail image of the study.

Finally, we performed a mediation analysis using β1, β2 and overall effect, to clarify the genetically estimated relationship between immune cell phenotypes and AF, mediated by plasma metabolites. We discovered that he 4-acetamidobutanoate levels mediated the relationship between CD25 on IgD + CD38dimand AF (*P* < .05). By employing the mediation analysis, the 4-acetamidobutanoate levels (mediated effect = 0.00157, *P*-value = .030) appear to mediate the pathway linking CD25 on IgD + CD38dim to AF (Table 4, Figs. 5 and 6).

**Table 4:** MR analyses of the causal effects between immune cells, plasma metabolites and AF.

Immune cell	Metabolite	Outcome	Mediated effect	*P*-value
CD25 on IgD + CD38dim	4-Acetamidobutanoate levels	AF	0.00157	.030
CD25 on IgD + CD38dim	Octadecanedioylcarnitine (C18-DC) levels	AF	0.00091	.142
CD25 on IgD + CD38dim	Linolenate [alpha or gamma; (18:3n3 or 6)] levels	AF	-0.00175	.053
CX3CR1 on CD14 + CD16- monocyte	N-Acetyl-aspartyl-glutamate (NAAG) levels	AF	-0.00132	.144

AF = atrial fibrillation, MR = Mendelian randomization.

## 4. Discussion

Our study demonstrated a causal relationship between 8 immune cell phenotypes and AF. The reverse MR analysis was performed to ensure the accuracy of our causal inference direction. For all *P* > .05 in reverse MR analysis, there were no indications of reverse causality from AF to 8 immune cell phenotypes. Through mediation analysis, we identified that 2 immune cell phenotypes could be causally associated with AF via 4 plasma metabolites. Notably, the levels of 4-acetamidobutanoate significantly mediate the pathway linking CD25 on IgD + CD38dim cells to AF.

Immune cells are important part of cardiovascular electrophysiological system and tissue homeostasis. Our MR analysis has identified 8 immune cell phenotypes with a causal relationship to AF, including IgD- CD38dim AC, CD4 + CD8dim AC, BAFF-R on IgD + CD38- naive, CD25 on IgD + CD38dim, CD27 on IgD- CD38br, CX3CR1 on CD14 + CD16- monocyte, CD64 on monocyte, and SSC-A on myeloid DC. In addition, we performed reverse MR with immunophenotype as the final phenotype and AF as a risk factor, which demonstrated that there was no significant association between these 2 and further improved the reliability of the results. It has been observed that the proportion of immune cells in cardiac tissues is about 4.7 ± 1.5% in healthy mice.^[36]^ Researchers have calculated the proportion of immune cells in different parts of the cardiovascular system. Specifically, myeloid cells accounted for 81.4 ± 1.4% approximately, B cells were 8.9 ± 0.6%, T cells were 3.1 ± 0.4% and non-myeloid/lymphoid immune cells were 6.6 ± 0.6% in ventricles.^[37]^ Another study of adult hearts showed that there were 11 major cell types, with bone marrow and lymphoimmune cells accounting for 10.4% of atrial tissue and 5.3% of ventricular tissue.^[38]^ In addition to the tissues in myocardium, there are some immune cells in the pericardial fluid and adipose tissues, which is important to local, even systemic immune response.^[39]^ Apart from the influence of distribution location, the plasticity of immune cells has also been shown to play a role in the immune regulation of AF. Immune remodeling has proved to induce inflammation and has relationship with atrial electrical, structural, and neural remodeling, which aggravate the potential value of immune regulation.

The classification of immune cells is complex, involving many biological functions and upstream and downstream relationships, establishing causality remains a significant challenge. Compared to other immunoglobulins (Ig), the function of IgD is less elucidated, which is thought to influence B-cell activity. If a B cell could produce a functional, non-autoreactive B-cell receptor, it differentiates into a mature and naive B cell.^[40]^ And then, these B cells express the B-cell receptor as IgM and IgD molecules, which is closely associated with diseases about immunity. In the study of zanubrutinib, a drug for the treatment of B-cell malignancies, many investigators have observed a variety of cardiovascular adverse effects, including AF.^[41]^ This reflects the potential impact of interventions targeting B-cell function on the cardiovascular system. Furthermore, the activation and maturity of B cells reflect the expression level of CD38. Several MR studies showed that IgD- CD38dim is related to sudden sensorineural hearing loss,^[42]^ systemic lupus erythematosus,^[43]^ and esophageal cancer.^[44]^ Increased levels of IgD- CD38dim absolute count were associated with a higher susceptibility to AF,^[45]^ which is consistent with our findings.

The expression of surface and intracellular markers helps researchers distinguish between different types of immune cells. The BAFF-receptor (BAFF-R) is encoded by the TNFRSF13C and is one of the main pro-survival receptors in B cells. The lack of BAFF-R leads to an almost complete block of B cell development and resulting immunodeficiency.^[46]^ Although CD25 is currently the common marker that could be used to purify functional Treg cells. It is an imperfect marker because it is also expressed on activated effector cells. Only CD27 could be used to identify Treg within these expanded cell lines of the markers reported to be stably expressed by Treg following activation.^[47]^ Some surface antigens are also not specifically expressed, but appear in different types of immune cells. A MR analysis discovered that membrane proteins on specific immune cells, such as CD25 on memory B cells-which functions as a part of the interleukin-2 receptor-may be risk factors for AF development.^[48]^ By demonstrating endothelial proatherosclerotic gene regulation in direct contact with CD16+ monocytes in part via cellular CX3CR1–CX3CL1 interaction, researchers showed that this cell type can increase cardiovascular risk.^[49]^ CD64 is an Fcγ receptor of immunoglobulin G that present on the surface of monocytes, participating and increasing in granulocytes in case of infection, systemic inflammatory response syndrome, or tissue damage.^[50]^ In our results, 5 immune cells showed a positive correlation with AF, including IgD- CD38dim AC, CD4+ CD8dim AC, CD27 on IgD- CD38br, CX3CR1 on CD14+ CD16- monocyte, and SSC-A on myeloid DC. While there is a negative correlation between 3 immune cell phenotypes and AF including BAFF-R on IgD+ CD38- naive, CD25 on IgD+ CD38dim, and CD64 on monocyte. Given the complexity and variability of immune cell functions, there are still many unclear fields that need more research to explore.

Immunometabolism has an impact on cardiovascular disease through inflammation.^[51]^ Plasma metabolite levels can provide clinical validity in the prediction of disease,^[52]^ which is an inspiration to explore new biomarkers. Our study showed a causal relationship between 4-acetamidobutanoate and AF, but no studies are addressing the link. 4-Acetamidobutanoate is a product of the urea cycle and amino acid metabolism, and a derivative of γ-aminobutyric acid (GABA), an inhibitory neurotransmitter of the nervous system.^[53]^ A cohort study has shown the use of gabapentin and pregabalin, GABA analogs, may increase the risk of AF in seniors.^[54]^ There is a case reported that a young African–American male developed new-onset AF after 4 days of gabapentin.^[55]^ This may explain the increased the risk of AF with 4-acetamidobutanoate level, but the exact mechanism is unknown. Otherwise, patients with obstructive sleep apnea have a higher incidence of AF than the general population,^[56]^ and dysregulation of neurotransmitters, including GABA, is thought to contribute to obstructive sleep apnea,^[57]^ and perhaps of concomitant AF. 4-Acetamidobutanoic acid represents a different chemical state of 4-acetamidobutanoate and exhibits a strong correlation with aging. A study involving 11,634 participants from the United States and 1878 participants from Spain demonstrated that elevated levels of 4-acetamidobutanoic acid were associated with increased all-cause mortality and reduced likelihood of longevity. Furthermore, the accumulation of 4-acetamidobutanoic acid intensifies with advancing age, heightening the risk of AF, which may contribute to the pathogenesis of this condition in the context of aging. 4-Acetamidobutanoate is also a product of nicotinamide adenine dinucleotide (NAD)-linked aldehyde dehydrogenase.^[58]^ Some immune cells, notably, CD38 on B cells has been proved to be associated with NAD metabolism. CD38 degrades extracellular NAD precursors and intracellular NAD, maintaining homeostasis of NAD levels in tissues.^[59–61]^ In addition to possibly promoting the production of 4-acetamidobutanoate, elevated NAD levels and its prolonged half-life in tissues increase the likelihood of accumulation of toxic metabolites such as nicotinamide adenine dinucleotide phosphate (NADPHX).^[62]^ In conclusion, these previous studies provide some basis for the causal relationship we obtained. This may be an explanation for the 4-acetamidobutanoate-mediated association of CD25 on IgD + CD38dim cells with AF, but it remains to be verified.

The other 3 metabolites, although shown to correlate with AF in MR analyses, do not support the hypothesis of a mediator between immune cell phenotype and AF. Linolenate [alpha or gamma; (18:3n3 or 6)], belongs to the group of polyunsaturated fatty acids, also been known as omega-3 and omega-6 fatty acids, which have been shown to have a protective effect on the heart.^[63]^ Current researches have found that controlling the Omega-6/Omega-3 ratio in the ideal range reduces the incidence of cardiovascular disease.^[64,65]^ Our study indicated that Linolenate [alpha or gamma; (18:3n3 or 6)] levels were negatively associated with a protective effect against AF, consistent with previous studies. NAAG is a neuropeptide that has shown to be protective against AF in the MR analysis. It is an antagonist of the N-methyl-D-aspartate receptor.^[66]^ An animal experiment has shown that activation of NMARD may increase susceptibility to AF.^[67]^ Octadecanedioylcarnitine (C18-DC) is a fatty acid metabolite associated with cellular energy metabolism pathways. It has been found that the C18-DC level elevated in patients with peroxisome biogenesis disorder.^[68,69]^ Although our MR analysis showed that C18-DC was a protective factor against AF, it has been poorly studied in cardiovascular disease.

Our study is the first to research the link between immune cells, circulating metabolites, and AF by bidirectional MR analysis. The extensive sensitivity analyses in the study also made the conclusions more reliable. Although we were able to identify the potential link from a wide range of immune cells and metabolites, and try our best to explore the 2 links have an exact effect on AF. In an attempt to prove causality, we use adapted Bradford Hill standards. For CD25 on IgD + CD38dim, 4-acetamidobutanoate levels, AF, most of the Bradford Hill criteria were fulfilled, including strength, analogy, consistency throughout the analyses, consistent findings across studies, temporal relationships, and biological plausibility, thus suggesting that there may be a causal relationship between them (Table 5). However, the study has certain limitations at present. Firstly, due to the complexity of immune regulation, the conclusions obtained from the study need to be validated and explored in depth in subsequent experimental and large-scale clinical studies. Secondly, since this study was conducted based on data from a European population, the conclusions obtained may not be fully applicable to other ethnic groups. Thirdly, due to the limitations of the data, we were unable to derive the effect of age and gender on the causal relationship.

**Table 5:** The criteria adapted from Bradford Hill to assess causality in Mendelian randomization study.

Criteria	Description	Source/method
Strength of the association	The larger the association, the more likely that it is causal. Verified causal relationships of immune cells, plasma metabolites, AF and considering only suspected mediation.	Five MR methods, sensitivity analysis and mediation analysis
Analogy	The immune cells and metabolic mediators were associated with a higher susceptibility to AF, or it is similar to pathogen known to induce it.	Literature search
Biological plausibility	Immune cells are integral components for maintaining homeostasis in cardiovascular system, both in the electrophysiological system and tissue. Immunometabolism and has an impact on cardiovascular disease through inflammation.	Literature search
Consistency	Consistent findings are observed on different analysis methods.	Five MR methods
Coherence	Coherent findings are observed when investigating the association of the same study with related results.	Literature search
Specificity	The association is specific for the immune cells and metabolic mediators considered and is not common to other immune cells and metabolic mediators.	Literature search and GWAS
Temporal relationship	We reverse the functions of exposure and outcome because exposure and outcome are a two-way street.	Bidirectional Mendelian randomization study
Reversibility	AF occurs or decreases if immune cells and metabolic mediators increase or decrease.	Literature search

AF = atrial fibrillation, MR = Mendelian randomization.

## 5. Conclusion

In summary, our MR study explored the relationship between immune cells, plasma metabolites, and AF. Finally, we identified causal relationships between 2 immune cell phenotypes and 4 plasma metabolites with AF. It’s worth noting that the 4-acetamidobutanoate levels appear to mediate the pathway linking CD25 on IgD + CD38dim to AF. This association could serve as valuable biomarker and potential target for prevention and treatment of AF.

## Acknowledgments

We thank all the participants and investigators involved in the GWAS to provide and share the original data.

## Author contributions

**Conceptualization:** Hongliang Huang.

**Data curation:** Hongliang Huang.

**Formal analysis:** Hongliang Huang.

**Investigation:** Hongliang Huang.

**Methodology:** Hongliang Huang.

**Software:** Hongliang Huang

**Visualization:** Hongliang Huang.

**Writing – original draft:** Hongliang Huang.
